# A Primary Mediastinal Large B-Cell Lymphoma Patient With COVID-19 Infection After Intensive Immunochemotherapy: A Case Report

**DOI:** 10.3389/fonc.2020.00924

**Published:** 2020-05-22

**Authors:** Qiuhui Li, Fang Zhu, Yin Xiao, Tao Liu, Xinxiu Liu, Gang Wu, Liling Zhang

**Affiliations:** Cancer Center, Union Hospital, Tongji Medical College, Huazhong University of Science and Technology, Wuhan, China

**Keywords:** COVID-19, lymphoma, myelosuppression, chemotherapy, immunotherapy

## Abstract

**Background:** The outbreak of coronavirus disease 2019 (COVID-19) had become a global public health event. Lymphoma patients need to be distinguished from the general population because of their deficient immune status and intensive anti-tumor treatment. The impacts of cancer subtypes and treatment on COVID-19 infection are unclear.

**Case Presentation:** We here report the case of a primary mediastinal large B-cell lymphoma patient who was infected with COVID-19 after intensive immunochemotherapy (DA-EPOCH-R). The patient developed a neutropenic fever during chemotherapy, and fever was persistent, although antibiotics were used. Initial chest CT was negative, and the patient received a throat swab test since the second CT showed evidence of pneumonia. With treatment with Arbidol Hydrochloride and LianHuaQingWen capsule, his COVID-19 was cured.

**Conclusions:** To the best of our knowledge, this is the first report focusing on COVID-19 infection in a lymphoma patient undergoing intensive immunochemotherapy. For those patients being treated with immunochemotherapy in epidemic areas, a reduced dose intensity of intensive chemotherapy should be considered, and the effect of immunotherapies such as rituximab on COVID-19 infection should be considered. The impacts of anti-cancer treatment on COVID-19 infection need to be explored further.

## Introduction

Primary mediastinal large B-cell lymphoma is a distinct subtype of non-Hodgkin lymphoma and has features that overlap with classic nodular sclerosing Hodgkin lymphoma (1). There is no standard treatment of primary mediastinal large B-cell lymphoma because of its rarity. DA-EPOCH-R (dose-adjusted etoposide, prednisone, vincristine, cyclophosphamide, doxorubicin, and rituximab) is a promising and effective regimen with an event-free survival rate of 93% and an overall survival rate of 97% during a median of 5 years of follow-up. However, grate 4 neutropenia occurred during 50% of cycles, and hospitalization for febrile neutropenia occurred during 13% of cycles (2).

Since December 2019, an epidemic of coronavirus disease 2019 (COVID-19) has broken out in Wuhan and spread across China and beyond. Patients with cancer might have an increased risk of COVID-19 and a poor outcome, as reported by Liang et al. (3). In Liang's report, only two (lung cancer patients) of 18 patients had received chemotherapy within the previous month. However, the effects of chemotherapy and immunotherapy on the risk of COVID-19 were not described. Here, we report a case of a patient who was infected with COVID-19 during the course of intensive immunochemotherapy.

## Case Description

A 26-year-old Chinese male patient presenting with a large mediastinal mass was diagnosed as having primary mediastinal large B-cell lymphoma (IPI 1 score). He denied a history of smoking or other diseases. He had finished two cycles of DA-EPOCH-R regimens and suffered febrile neutropenia each time. He was admitted for the third cycle of chemotherapy on January 9, 2020. Physical examination revealed that the swelling in the face, neck, and upper limbs was reduced, but distention of the jugular vein was still visible. Also, enlarged lymph nodes in the cervical and supraclavicular areas returned to normal size and were not palpated after two cycles of therapy. He was evaluated as being in partial remission on the basis of contrast CT.

**Figure 1:**
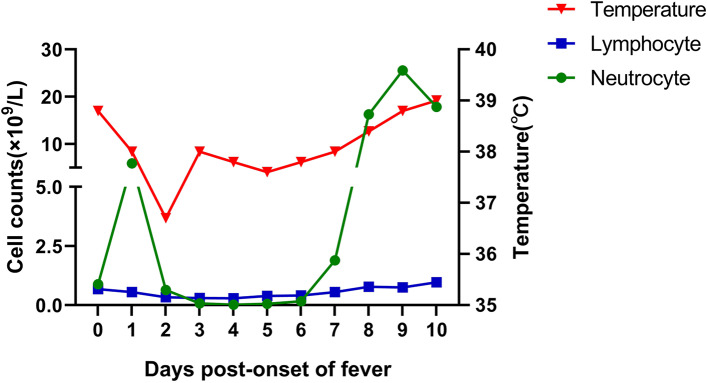
Changes in maximum body temperature and blood cell count after the onset of fever.

**Figure 2:**
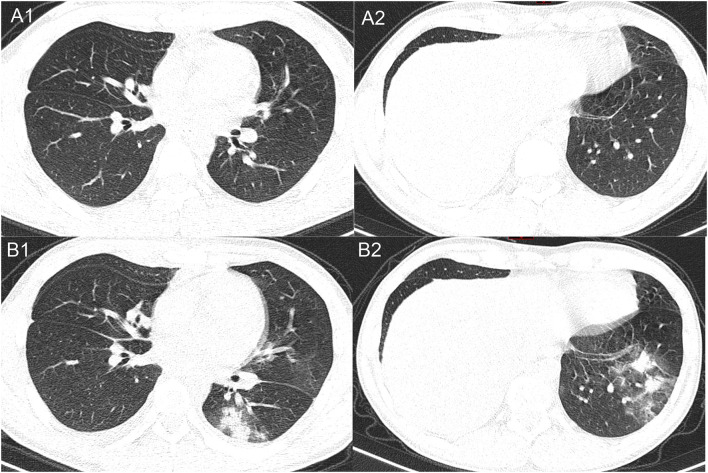
Representative images of the chest CT comparing between January 19, 2020 **(A1, A2)**, and January 27, 2020 **(B1, B2)**. The later CT images show multifocal opacities and consolidations in different lobes of the lungs.

With no obvious abnormalities in CT images and laboratory examinations, the patient was administrated the third cycle of DA-EPOCH-R as planned from January 12 to January 17. On January 19, the patient developed a fever (38.8°C) without cough, dyspnea, myalgia, or fatigue. His neutrocyte count was 0.89 × 10^9^ cells/L, his lymphocyte count was 0.68 × 10^9^ cells/L, and chest CT showed no evidence of infection. We treated the patient for febrile neutropenia using antibiotics (Meropenem and Linezolid) and granulocyte colony-stimulating factor (G-CSF). However, fever was persistent, and grade 3/4 neutropenia remained from January 21 to January 25, 2020 (Figure 1). Further examination revealed that antibodies of *Mycoplasma pneumoniae* IgM, coxsackie B5 virus IgM, and enterovirus RNA were positive, and procalcitonin was normal. Thus, Azithromycin and Ganciclovir were applied, and Oseltamivir and Posaconazole were also applied to prevent the influenza virus and fungal infections. On January 27, the neutrophils returned to normal, but the patient complained of sore throat and nausea and had a fever of 38.4°C. A repeated chest CT revealed bilateral scattered opacities and consolidation; bilateral pleural effusion and segmental atelectasis were also seen (Figure 2). Consultation with the infectious disease expert group suggested that a mixed infection could be in existence, and SARS-CoV-2 infection should be suspected. Therefore, an RT-PCR test for SARS-CoV-2 was performed, and the patient was confirmed to have COVID-19 infection. He was transferred to a designated hospital on January 30. With the treatment of oral Arbidol Hydrochloride (0.2 g per time, three times a day for 1 week) and LianHuaQingWen capsule (1.4 g per time, three times a day for 10 days), his RT-PCR tests became negative, and CT images improved. He was considered cured and was discharged on February 17. He did not develop severe pneumonia during treatment and did not need a ventilator to help him breathe.

## Discussion

Lymphopenia is a common laboratory finding in confirmed COVID-19 cases; it was present in 83.2% of the patients on admission (4). It is also a key item recommended for clinical suspicion of a case according to the Diagnosis and Treatment Program of COVID-19 (trial Seventh version) issued by China's National Health Commission in March 2020. However, intensive chemotherapy can cause severe neutropenia and lymphopenia. Therefore, the lymphocyte count cannot be regarded as a key point upon which SARS-CoV-2 infection can be suspected in cancer patients who are undergoing intensive chemotherapy and suffering grade 3/4 hematologic toxicity.

In the early stage of the COVID-19 epidemic, chest CT scan plays a vital role in early diagnosis. As radiologists have described, COVID-19 pneumonia typically manifests with rapid evolution from focal unilateral to diffuse bilateral ground-glass opacities that progress to or co-exist with consolidations in chest CT imaging. “White lung” may be seen in critically ill patients, and, rarely, patients may develop pleural effusion (5, 6). However, the initial CT scan of this patient since the onset of fever did not show any typical manifestations of COVID-19, suggesting that some COVID-19 cases take a few days to show significant CT changes. Indeed, several studies have revealed that 3.5–19% of laboratory-confirmed COVID-19 cases had a negative initial CT before progressing to pneumonia 3–5 days later (4, 6, 7).

Severe myelosuppression from intensive chemotherapy may increase risk of infection by bacteria, viruses, and fungi (8). For this patient, biomarkers reflecting infections by other pathogens, such as *M. pneumoniae*, coxsackie B5 virus, and enterovirus, were positive. Therefore, even when there was evidence of other viral infections and CT findings were atypical, a co-infection with SARS-CoV-2 should be considered and tested for. We should consider that the cases of cancer patients undergoing intensive chemotherapy and with confirmed SARS-CoV-2 infection could be complicated by other pathogen infections.

The first report focusing on COVID-19 and cancer indicated that patients with cancer may have worse outcomes (3). This patient's COVID-19 infection occurred when he was experiencing grade 3/4 hematologic toxicity after intensive immunochemotherapy. Thus, it was suspected that he might develop severe pneumonia because of the immunosuppression induced by immunochemotherapy. However, the course of this patient's illness seems to contradict this conclusion. Actually, other researchers have raised questions about Liang's conclusions (9, 10). They pointed out that the current evidence to support the conclusion that cancer patients have a higher susceptibility to SARS-CoV-2 and poorer prognosis remains insufficient. They also suggested that older age and smoking might be the true factors associated with worse COVID-19 outcomes (9–11). Whether the blunted immune status of cancer promotes or inhibits the development of COVID-19 infection is not clear. Therefore, larger samples and well-designed studies are needed to elucidate the relationship between cancer and COVID-19.

Rituximab is a chimeric monoclonal antibody directed against CD20-positive surface antigens on B lymphocytes. Rituximab induces a rapid and prolonged depletion of CD20-positive B lymphocytes and thus targets the cells that are key to successful immunization (12). Recently, although there is not enough evidence to stop using rituximab in lymphoma patients, some suggestions have been proposed by the American Society of Hematology (ASH) and the European Society for Medical Oncology (ESMO). The ASH website suggests trying to avoid or skip treatment with monoclonal antibodies (rituximab, obinutuzumab), especially when given in combination with targeted agents for chronic lymphocytic leukemia/small lymphocytic lymphoma (CLL/SLL) (13). On the other hand, rituximab maintenance may be delayed for mantle cell lymphoma and follicular lymphoma patients, as suggested by the ASH and ESMO (14, 15). However, rituximab is still recommended for diffuse large B-cell lymphoma patients (16). Decisions to use rituximab should thus be made on a case-by-case basis.

So far, both RNA vaccine in the United States and recombinant vaccine (adenovirus vector) in China targeting SARS-CoV-2 have entered phase I clinical trial (17, 18). It can be predicted that SARS-CoV-2 vaccines will be available in the near future. Previous studies have shown that patients undergoing rituximab-containing treatment regimens within the past 6 months do not respond to influenza A vaccination (19, 20). Whether the application of rituximab will affect the production of antibodies to SARS-CoV-2 or the effect of the SARS-CoV-2 vaccination is worth exploring further.

Our case illustrates the first reported case of COVID-19 infection in a lymphoma patient who underwent intensive immunochemotherapy and exhibited grade 4 myelosuppression. Since COVID-19 was declared a pandemic on March 11, 2020, lymphoma patients worldwide face risk of infection. For those patients being treated with immunochemotherapy in epidemic areas, reduced dose intensity of intensive chemotherapy should be considered, and the effect of immunotherapy, such as rituximab, on COVID-19 infection should be considered. The impacts of anti-cancer treatment on COVID-19 infection need to be explored further.

## Data Availability Statement

The raw data supporting the conclusions of this article will be made available by the authors, without undue reservation.

## Ethics Statement

Written informed consent was obtained from the patient for the publication of any potentially identifiable images or data included in this article.

## Author Contributions

QL and LZ wrote and edited the final manuscript. GW, LZ, and QL designed the study and managed the patient's care at the cancer center. FZ, YX, TL, and XL assisted in data collection, data analysis, interpretation, and construction of figures. All authors read and approved the final manuscript.

## Conflict of Interest

The authors declare that the research was conducted in the absence of any commercial or financial relationships that could be construed as a potential conflict of interest.
